# Aerosol Gemcitabine after Amputation Inhibits Osteosarcoma Lung Metastases but Not Wound Healing

**DOI:** 10.1155/2018/3143096

**Published:** 2018-01-21

**Authors:** Eugenie S. Kleinerman, Ling Yu, Jasmine Dao, Andrea A. Hayes-Jordan, Brock Lindsey, Jitesh D. Kawedia, John Stewart, Nancy Gordon

**Affiliations:** ^1^The University of Texas MD Anderson Cancer Center, Division of Pediatrics, 1515 Holcombe Blvd., Unit 0853, Houston, TX 77030, USA; ^2^The University of Texas MD Anderson Cancer Center, Stem Cell Transplantation Research, 1515 Holcombe Blvd., Houston, TX 77030, USA; ^3^Nightlight Urgent Care, 15551 Southwest Freeway, Sugar Land, TX 77478, USA; ^4^Department of Surgical Oncology, The University of Texas MD Anderson Cancer Center, Children's Cancer Hospital, 1515 Holcombe Blvd., Unit 1484, Houston, TX 77030, USA; ^5^Department of Orthopedic Surgery, University of West Virginia, P.O. Box 9196, Morgantown, WV 26506-9196, USA; ^6^The University of Texas MD Anderson Cancer Center, Pharmacy Pharmacology Research, 1515 Holcombe Blvd., Unit 0090, Houston, TX 77030, USA; ^7^Department of Pathology, The University of Texas MD Anderson Cancer Center, 1515 Holcombe Blvd., Houston, TX 77030, USA

## Abstract

**Background:**

In newly diagnosed osteosarcoma (OS) patients, the time between surgery and resumption of chemotherapy is 2–7 weeks. Delays > 16 days are associated with increased risk of relapse and decreased overall survival. Identifying an effective therapy that can be used postoperatively may prevent relapse. We investigated whether aerosol gemcitabine (GCB) initiated after tumor resection inhibited the growth of OS lung metastases without affecting the wound-healing process.

**Methods:**

Mice were injected intratibially with OS cells. Amputation was performed when the tumor reached 1.5 cm. Full-thickness excisional wounds were also made on the dorsal skin and tail. Aerosol GCB or PBS was initiated 48 hours after amputation (3 times/week for 3 weeks). Wound sections were evaluated by immunohistochemistry for Ki-67 (proliferation), CD31 (vessels), VEGF, IL-10, bFGF, mast cells, macrophages, and M1/M2 macrophage ratios. The lungs were analyzed for macro- and micrometastases.

**Results:**

Aerosol GCB inhibited the growth of the lung metastases but had no effect on the 3 phases of wound healing in the dorsal skin, tail, or bone. Production of cytokines at the wound sites was the same.

**Conclusion:**

These data indicate that initiating aerosol GCB postoperatively may kill residual lung metastases thereby preventing relapse and improve survival.

## 1. Introduction

Osteosarcoma is the most common malignant bone tumor in both adults and children. While the introduction of combination chemotherapy given in addition to surgery improved the overall survival from 20 to 65%, survival rates have remained stagnant for >25 years [1–3]. The lung is most common site of metastatic spread, and the majority of newly diagnosed patients have undetectable microscopic lung metastases at the time of diagnosis providing the rational for giving preoperative and postoperative chemotherapy. Despite the use of aggressive combination chemotherapy, the development of visible lung metastases continues to be the major challenge in curing this disease. For patients who develop relapsed disease in the lung, the 5-year survival rate is only 20% [4, 5], and second-line chemotherapy has not made a significant impact on improving this outcome [6]. It is therefore critical to eradicate these micrometastases during the initial treatment period.

Prior to surgery, systemic chemotherapy serves to control the growth and size of the primary tumor to decrease the morbidity of surgery in addition to targeting the microscopic disease in the lung. Chemotherapy is stopped a few weeks before and for several weeks after surgery due to the interference of systemic chemotherapy on wound healing. Once patients recover from surgery, chemotherapy is resumed to prevent macroscopic metastases from developing. This results in a break or delay in chemotherapy administration, leaving patients untreated for a significant period of time when tumor rebound is possible. It is therefore important that this delay be as short as possible. The standard time between the surgery and the resumption of chemotherapy treatment is 2–4 weeks, but delays as much as 6–8 weeks have been reported in patients with postoperative complications such as infection or other comorbidities that lead to delayed healing [7–9]. Delays > 16 days have been associated with an increased risk of relapse and a significant decrease in overall survival [7, 8]. With 21–40% of patients experiencing delays [7, 8], identifying an effective “bridge therapy” against osteosarcoma lung metastases which does not interfere with postoperative healing and can be used in the immediate postoperative period has the potential to prevent relapse and increase both the event-free and overall survival.

Wound healing is complex and includes 3 distinct phases [10, 11]. First is the inflammatory phase which consists of the recruitment of inflammatory cells, including neutrophils, mast cells, and macrophages, both M1 and M2. M2 macrophages promote cell proliferation and tissue repair, while M1 macrophages inhibit cell proliferation. The second phase involves the migration and proliferation of fibroblasts and endothelial cells [12, 13]. Tissue remodeling is the third phase. Several specific cytokines and growth factors are critical to the healing process including IL-10 and bFGF [14–17]. A potential bridge therapy candidate should not interfere with any of the three wound-healing phases.

Gemcitabine (GCB) is a deoxycytidine analogue that causes DNA damage, initiating cell death [18]. GCB given systemically together with Taxol has shown modest activity against relapsed large lung metastases [19]. The activity of aerosol delivery to target OS lung metastases has been investigated. Aerosol therapy has the advantage of delivering agents directly to the lung, the organ where micrometastases exist, resulting in increased drug concentration in the lung, decreased drug levels in the circulation, and decreased systemic toxicity [20]. Using 1/10th the systemic dose, the effect of *aerosol* GCB against human and mouse OS lung metastases *in vivo* was demonstrated [21, 22]. Maximum therapeutic activity was seen against microscopic disease. Peak serum levels following 1.0 mg/kg aerosol GCB were 200 ng/ml, significantly lower than the peak serum levels (700 ng/kg) following 1.0 mg/kg GCB given systemically. No liver, kidney, lung, or hematologic toxicity was observed in the mice following 5 weeks of aerosol GCB given 3 times/wk. In addition, aerosol GCB demonstrated significant therapeutic benefit in dogs with visible osteosarcoma lung metastases [23]. Finally, Phase I/II trials using aerosol GCB demonstrated that this therapeutic approach is safe with no significant toxicity in regard to organ function or hematologic effects [24]. Taken together, these studies indicate that aerosol GCB may be a candidate for bridge therapy between the surgery and the restarting of systemic chemotherapy. However, there are no data in mice or patients on whether aerosol GCB affects wound healing.

We therefore wished to determine whether aerosol delivery of GCB is effective against OS lung metastases without interfering with wound healing in the skin and the bone following limb amputation. We evaluated the *in vivo* effect of aerosol GCB initiated 48 hours after amputation of a primary OS tumor in the tibia on established OS lung metastases and the healing of bone, dorsal skin, and tail wounds. While aerosol GCB successfully eradicated the microscopic lung metastases, aerosol GCB had no significant effect on the *in vivo* wound-healing process.

## 2. Materials and Methods

### 2.1. Cell Lines

K7M3 murine osteosarcoma metastatic cells [21] were cultured in Dulbecco's modified Eagle's medium (DMEM) and supplemented with 10% fetal calf serum (HyClone, USA) and 1% penicillin/streptomycin (Lonza, USA). The cells were maintained at 37°C in a humidified atmosphere of 5% carbon dioxide in air and harvested at 80–90% confluency.

### 2.2. Animal Model

BALB/c mice (Charles River, USA) were anesthetized with isoflurane. K7M3 cells suspended in 10 *µ*l of sterile phosphate-buffered saline (PBS) at a density of 1 × 10^6^ cells were injected into the tibia as previously described [22, 25]. Local tumors were detected at 3-4 weeks after injection, and the metastases were visible by 5-6 weeks [25].

### 2.3. Amputation

When tumor size reached 1.5 cm in diameter, the mouse was anesthetized with isoflurane and the leg was amputated above the knee joint with a sterile blade followed by the application of surgical staples. To minimize the postoperative pain, buprenorphine (0.1 mg/kg) was given as s.c. injection every 8–12 hours. The amputation wound was monitored thereafter during the aerosol PBS and GCB treatment.

### 2.4. Preparation of Wound Tissue

Mice were also used in the dorsal cutaneous and tail wound experiments one day after amputation. The mice were anesthetized with isoflurane, and the dorsal cutaneous skin was shaved and wiped with 70% ethanol. Full-thickness excisional wounds were made by picking up a fold of skin using a sterile disposable 6 mm biopsy punch, resulting in the generation of one wound on each side of the midline [26, 27]. A separate tail wound 10 mm × 3 mm was made on the tail of each mouse down to the fascia on the same day as the dorsal wound. The two wounds were photographed every 3 days, and dimensions were measured every 3 days, particularly noting reepithelialization of the skin [28].

### 2.5. Aerosol GCB Treatment

Ten mice were treated with aerosol PBS (control) or aerosol GCB at 1.0 mg/kg in 10 ml saline. For comparison of serum GCB concentration, 10 mice were treated with intraperitoneal injection of 1.0 mg/kg GCB in 0.2 ml of normal saline 3 times weekly for 3 weeks as previously described [22]. Blood was collected in tubes containing 0.75 mg of tetrahydrouridine, a cytidine deaminase inhibitor, to prevent *ex vivo* metabolism of gemcitabine. Blood samples were processed to separate plasma and kept at −80°C until analysis. Serum was collected at 10 min, 30 min, and 24 hours after GCB treatment. Samples were sent to Pharmacy Pharmacology Research Department for analysis of the serum GCB level.

### 2.6. Histology and Immunohistochemistry

Tissues from the amputation wound and dorsal cutaneous and tail wounds were collected on days 7, 14, and two months after the surgery and aerosol GCB treatment. Tissue sections were formalin fixed and paraffin embedded. Decalcification was performed for the amputation wound.

Mast cell numbers were quantified using toluidine blue staining and counting the number of toluidine blue positive cells in 10 high-power field (h.p.f) under a 20x objective lens.

The total number of macrophages was quantified using anti-F4/80 antibody staining (ab6640, Abcam USA). M1 macrophages were identified and quantified using iNOS staining (PA5-16855, Thermo Fisher Scientific). Anti-mannose receptor antibody (ab 64,693, Abcam USA) was used to identify and quantify M2 macrophages. CD31 antibody (ab 28,364, Abcam USA) was used for endothelial cell staining; anti-VEGFR-2 (ab39256, Abcam USA) was used for VEGF staining; IL-10 and FGF-2 were measured using ab189392 (Abcam USA) and sc-1360 (Santa Cruz Biotechnology), respectively. Ki67 antibody (ab15580, Abcam USA) was used for cell proliferation and fibroblast activation; protein alpha antibody (ab53066, Abcam USA) was used for fibroblast staining. All the sections were incubated with the primary antibody overnight at 4°C in accordance with the protocols provided by companies.

### 2.7. Statistics

To determine the significance between two groups, we used GraphPad Prism. The statistical analysis between two groups was performed using the unpaired Student's *t*-test. *P* > 0.05 was considered to have no significant difference.

## 3. Results

### 3.1. Serum GCB Levels following Aerosol versus Intraperitoneal Administration

**Figure 1:**
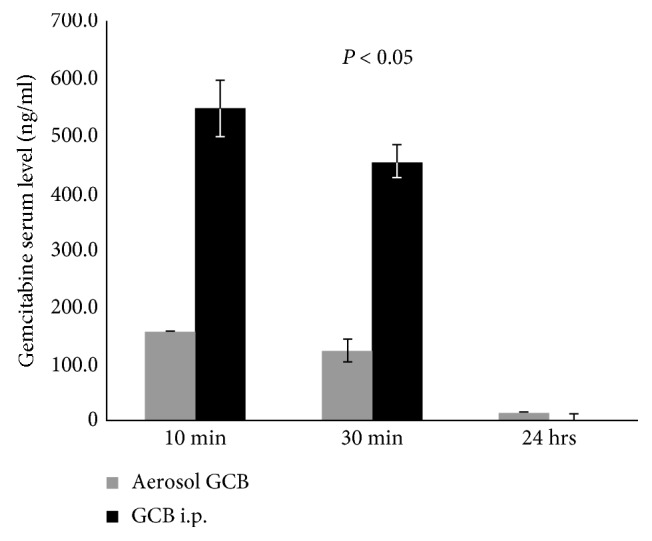
Serum GCB levels following intraperitoneal (i.p.) and aerosol GCB. Mice were treated with 1 mg/kg GCB given i.p. or by aerosol administration. Blood was collected at various times following administration, and serum GCB levels were quantified. Mice treated with aerosol GCB had significantly lower serum levels (*P* < 0.05).

We first compared the serum levels of GCB following aerosol or intraperitoneal administration. Serum GCB levels in the mice treated with GCB i.p. were 3.5-fold higher than those treated with aerosol GCB at 10 min and 30 min after administered (Figure 1).

### 3.2. Aerosol GCB Had No Effect on Wound Healing but Inhibited Osteosarcoma Lung Metastases

**Figure 2:**
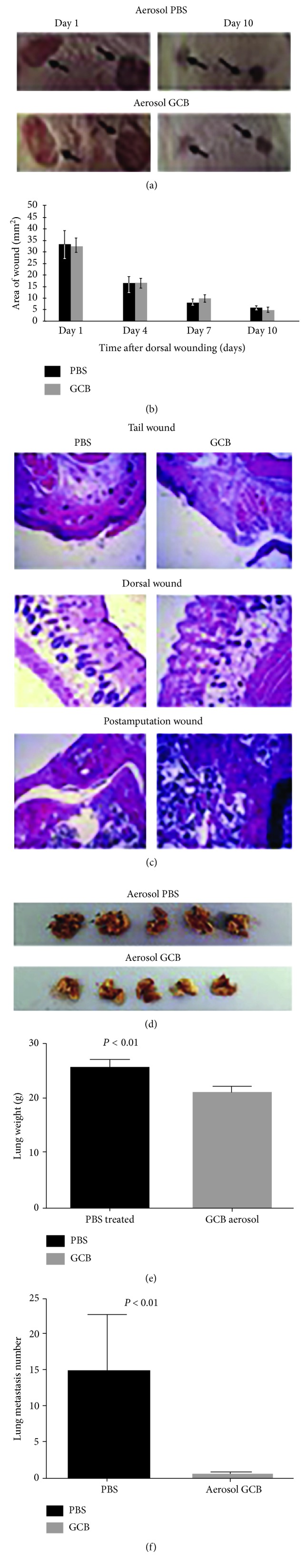
Aerosol GCB inhibited lung metastases but had no effect on wound healing in the tail, dorsal skin, or bone. (a) Representative appearance of the dorsal wound on days 1 and 10 in the mice treated with aerosol PBS (top row) or aerosol GCB (bottom row). (b) The areas of each wound in the aerosol PBS and aerosol GCB groups were measured 1, 4, 7, and 10 days after treatment (*P* > 0.05 for each group). (c) Representative H&E sections from the wounded tail, dorsal skin, and postamputation area 7 days following wounding. (d–f) Aerosol GCB inhibited the growth of osteosarcoma lung metastases as assessed visually (d), by the lung weight (e), and by the mean number of lung metastases (f) (*P* < 0.01).

In order to mimic the clinical course of osteosarcoma, K7M3 cells were injected into the tibia. Micrometastases in the lung were confirmed by sacrificing 2 mice 3 weeks later. When the tumors measured 1.5 cm, tail and dorsal skin wounding and amputation of the affected limb were performed. The mice were divided into 2 groups and treated with either aerosol PBS or aerosol GCB 24 hours after amputation. Aerosol therapy continued twice a week for three weeks. Healing of the tail, dorsal skin, and amputation site was monitored. Skin wounds were measured 4, 7, and 10 days after the initiation of aerosol therapy. There was no difference in wound healing of the tail or dorsal skin between the aerosol PBS- and aerosol GCB-treated mice (Figures 2 and 2). Similar to dorsal and tail wounds, aerosol GCB also did not affect the healing of the amputation site as shown by H&E staining in 3 different regions (Figure 2).

To determine whether aerosol GCB inhibited lung metastasis, five mice from each treatment group were sacrificed after 3 weeks of aerosol therapy. Aerosol GCB significantly decreased the number of visible lung metastases compared to the aerosol PBS group (Figure 2). Lung weights and the number of micrometastases were also significantly reduced in the aerosol GCB group (Figures 2 and 2).

### 3.3. Effect of Aerosol GCB on Cell Proliferation and the Number of Fibroblasts in the Wound Areas

**Figure 3:**
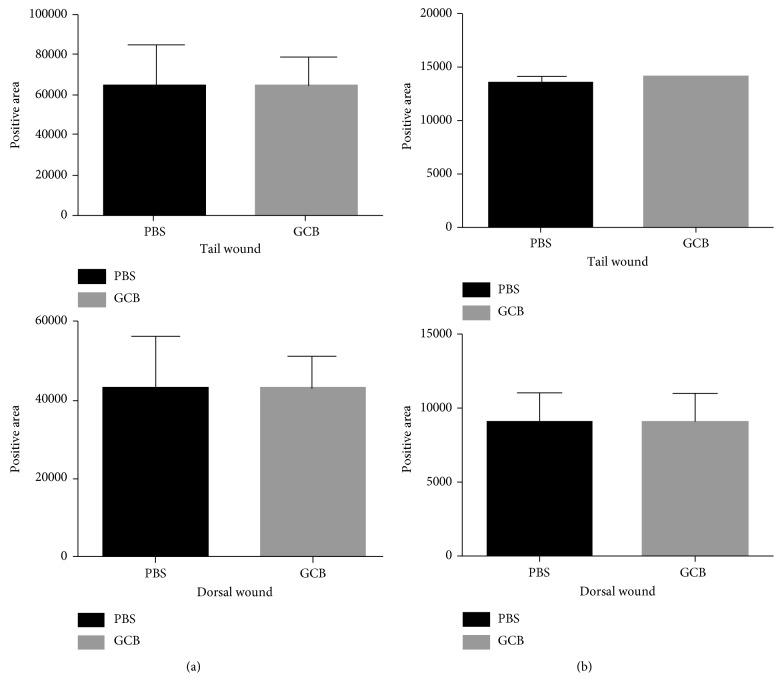
Effect of aerosol GCB on cell proliferation and fibroblast numbers associated with wound healing. The dorsal and tail skin wounds from the mice treated with aerosol PBS or aerosol GCB were examined on day 7. (a) Cell proliferation was assessed using Ki67. (b) Fibroblast numbers were assessed using anti-fibroblast antibody. The positive areas were quantified by SimplePCI groups obtained from five h.p.f areas and compared using Student's *t*-test. *P* > 0.05 for all graphs.

Ki67 is a marker of cell proliferation. We therefore quantified the number of Ki67^**+**^ cells in the tail and dorsal skin wounds 7 days after treatment with aerosol therapy. There was no difference in the positive areas for Ki67 staining between aerosol PBS and aerosol GCB (Figure 3, *P* > 0.05 for both dorsal and tail wounds). In addition, quantification of the positive areas for fibroblasts using immunohistochemistry also showed no difference between aerosol PBS and aerosol GCB (Figure 3, *P* > 0.05 for both dorsal and tail wounds). These results demonstrate that aerosol GCB had no effect on cell proliferation or the number of fibroblast cells during wound healing.

### 3.4. Aerosol GCB Did Not Affect the 3 Critical Phases of Wound Healing

**Figure 4:**
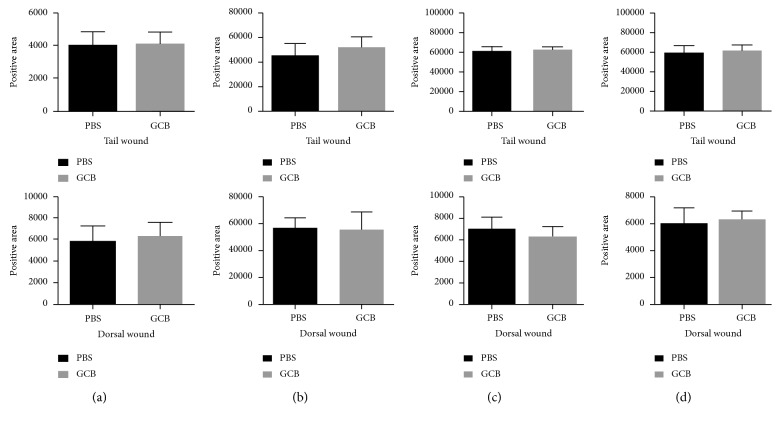
Effect of aerosol GCB on mast cell and macrophage infiltration and M1/M2 wound content. The dorsal and tail skin wounds from the mice treated with aerosol PBS or aerosol GCB were examined on day 7 for (a) mast cells using toluidine blue and (b) macrophage content using F4/80 antibody. (c) M1 macrophages were identified by anti-iNOS; (d) M2 macrophages were identified using anti-mannose receptor. The positive areas were quantified by SimplePCI groups obtained from five h.p.f areas and compared using Student's *t*-test. *P* > 0.05 for all graphs.

Wound repair typically consists of three phases: initial inflammation, followed by proliferation and finally remodeling. To determine whether aerosol GCB affects wound-induced inflammation, we quantified the number of mast cells in the wound tissues using toluidine staining, the number of macrophages using F4/80 antibody staining, and the ratio of M1 to M2 macrophages. M1 macrophages, which inhibit cell proliferation and cause tissue damage, were identified and quantified using iNOS staining. M2 macrophages, which promote cell proliferation and tissue repair, were identified and quantified using the anti-mannose receptor antibody. Both types of macrophages are important for normal wound healing as is the M1/M2 ratio. Aerosol GCB had no effect on the number of mast cells (Figure 4, *P* > 0.05) or the number of M1 and M2 macrophages (Figure 4, *P* > 0.05) in both tail and dorsal wounds. In addition, we showed that the M1/M2 ratio in the tail and dorsal wounds did not differ between aerosol PBS- and aerosol GCB-treated mice (M1 Figure 4, *P* > 0.05; M2 Figure 4, *P* > 0.05). These results demonstrated that aerosol GCB does not affect the first phase of wound healing, that is, wound-induced inflammation in the mice.

We next determined whether aerosol GCB affected endothelial cell proliferation and angiogenesis, both critical functions in the second phase of wound healing.

Endothelial cells play an important role in angiogenesis in wound-healing process.

**Figure 5:**
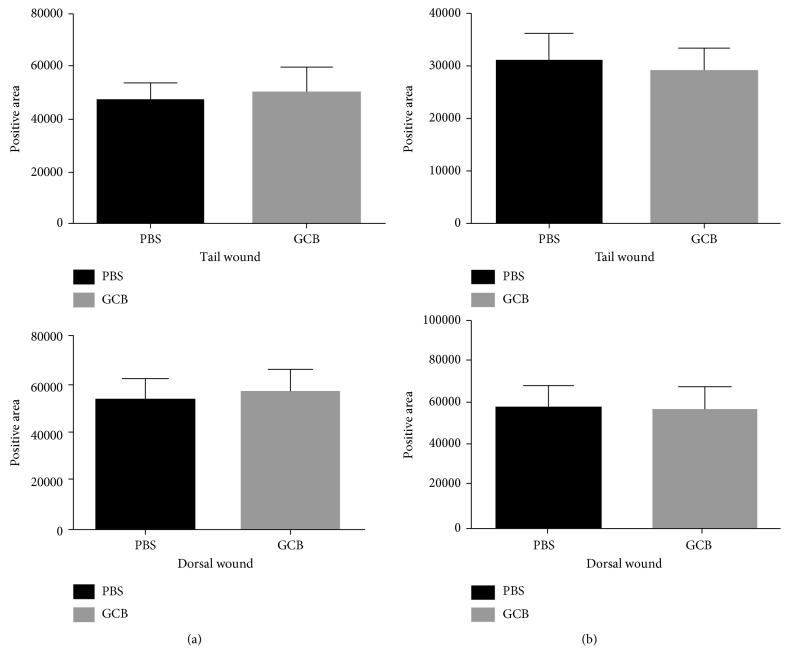
Effect of aerosol GCB on CD31 and VEGFR in the wound area. The dorsal and tail skin wounds from the mice treated with aerosol PBS or aerosol GCB were examined on day 7 using (a) anti-CD31 or (b) anti-VEGFR The positive areas were quantified by SimplePCI groups obtained from five h.p.f areas and compared using Student's *t*-test. *P* > 0.05 for all graphs.

To explore whether GCB affects endothelial cell growth and recruitment in wound healing, we quantified the number of CD31^**+**^ cells (a marker expressed by endothelial cells) in dorsal and tail wound tissues 7 days after aerosol PBS or aerosol GCB. There was no difference in CD31 staining in the tail and dorsal skin wounds between aerosol PBS- and aerosol GCB-treated mice (Figure 5, *P* > 0.05). VEGFR-2 has been shown to mediate almost all of the known cellular responses to VEGF [29]. We therefore measured the density of VEGFR-2^**+**^ cells in the wound areas and once again found no difference between the aerosol PBS- and aerosol GCB-treated groups (Figure 5, *P* > 0.05). These results indicate that aerosol GCB did not inhibit angiogenesis (an important process in wound dealing) or interfere with the growth of endothelial cells.

To determine whether aerosol GCB affected tissue remodeling (the third phase of wound healing), we quantified IL-10 and FGF-2 (bFGF), both of which are critical cytokines for the remodeling phase of wound healing.

**Figure 6:**
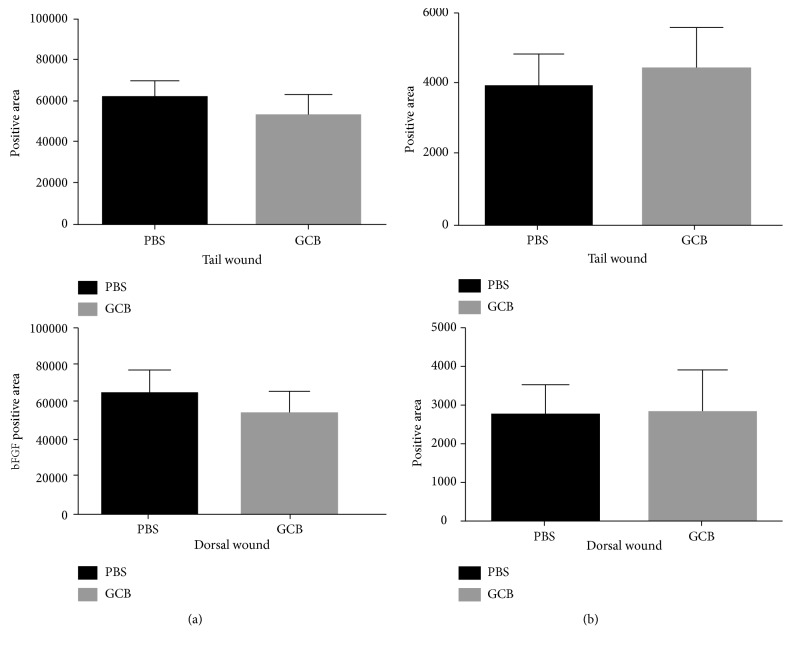
Effect of aerosol GCB on IL-10 and FGF-2 (bFGF) in the wound. The dorsal and tail skin wounds from the mice treated with aerosol PBS or aerosol GCB were examined on day 7 using (a) anti-IL-10 or (b) anti-FGF-2. The positive areas were quantified by SimplePCI groups obtained from five h.p.f areas and compared using Student's *t*-test. *P* > 0.05 for all graphs.

IL-10 plays an important role in wound healing through its function to inhibit the infiltration of neutrophils and macrophages toward the site of the wound area during this phase [28]. We measured the positive areas of IL-10 in dorsal wound and tail wound sections 7 days after GCB treatment. Again, we showed no difference in tail or dorsal wounds (Figure 6, *P* > 0.05) between aerosol GCB- and aerosol PBS-treated mice. FGF-2 or bFGF mediates the formation of new blood vessels that are critical in the wound-healing process following surgery. Different studies demonstrated a correlation between reduced FGF-2 expression and wound-healing disorders [16, 30, 31, 33]. mRNA levels of FGF-2 were reduced during wound healing in healing-impaired genetically diabetic mice compared with control mice [38]. Expression of FGF-2 was found to be upregulated after injury in normal but not in diabetic rats. Impaired would healing was seen in aged mice, and this impairment was associated with reduced levels of FGF-2 [33]. Finally, when FGF-2 null mice were used for wound-healing studies, they showed delayed healing, while there was no delay seen in FGF-1-knockout mice [30, 31] Similar to our findings with IL-10, there was no difference in the expression of FGF-2 (bFGF) in the dorsal and tail wounds between the mice treated with aerosol PBS and aerosol GCB (Figure 6, *P* > 0.05).

### 3.5. Effect of Aerosol GCB on Wound Healing following Amputation

**Figure 7:**
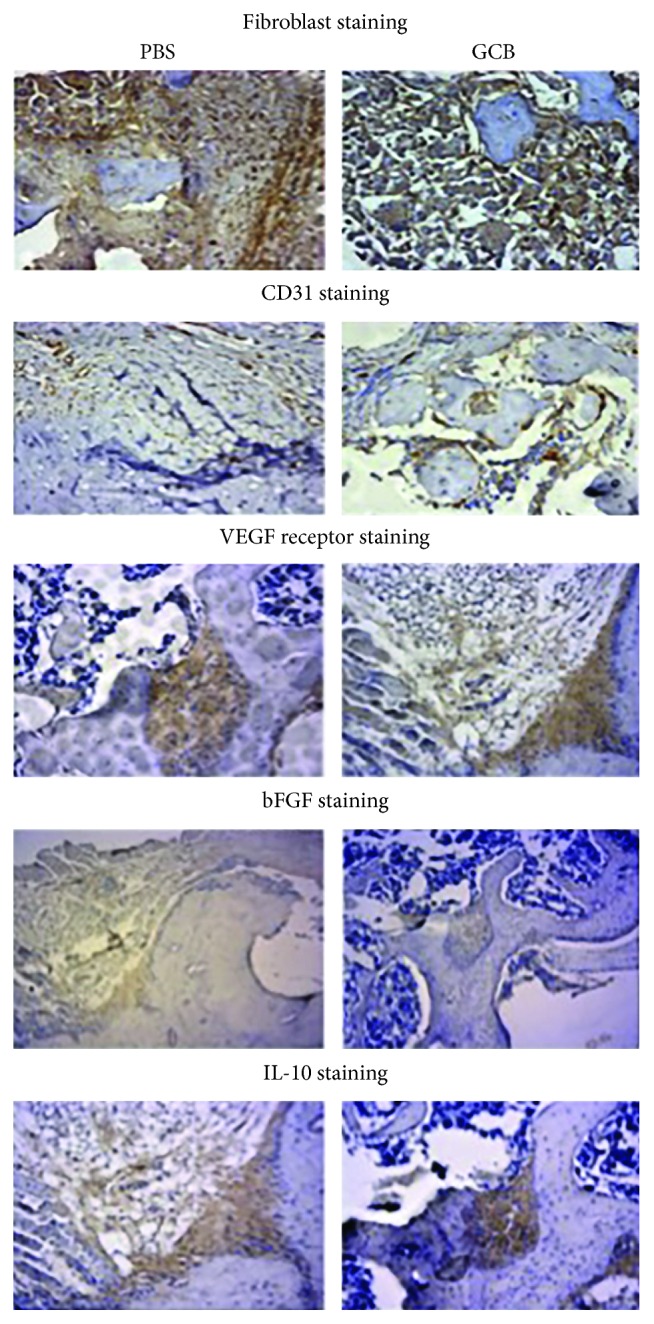
Aerosol GCB did not affect bone healing following amputation. Representative sections of immunohistochemistry of the mice 14 days after amputation for fibroblasts, CD31, VEGFR-2, FGF-2 (bFGF), and IL-10.

Healing of the bone and tissue following amputation and tumor removal is a critical part of the patient recovery process. We therefore monitored healing of the amputated area every other day. The amputation wound-healing process in the mice following aerosol GCB administration was not delayed compared with the mice treated with aerosol PBS. Similar to what we observed in the tail and dorsal skin wounds, there was no difference in fibroblast proliferation, CD31 expression, VEGFR-2 expression, or the wound-associated cytokines FGF-2 (bFGF) and IL-10 between aerosol PBS and GCB groups (Figure 7).

## 4. Discussion

Combination chemotherapy given both pre- and postoperatively has raised the overall survival of patients with primary nonmetastatic osteosarcoma from 20 to 65% [1–3]. However, despite the use of aggressive multiagent chemotherapy, 30–35% of patients who have no detectable metastasis at the time of diagnosis develop pulmonary metastases following surgical resection and adjuvant chemotherapy. This statistic has not improved in >30 years, and patients who develop lung metastases have a significantly reduced long-term survival [4, 5]. Equally disturbing is that there has been relatively little success in treating relapsed patients with surgical resection of metastases in the lung, which is the most effective approach [32]. A recent analysis showed that the median time to progression in multiple Phase II trials in the Children's Oncology Group for children and adolescents with relapsed osteosarcoma was ∼4 months [34]. Furthermore, no responses were seen in multiple Phase I trials from a single institution [35]. Due to the absence of effective secondary agents and the poor response rates to date for relapsed patients, it is critical to identify conditions that put the patients at a higher risk for relapse.

Postoperative chemotherapy is usually not initiated for 2–8 weeks after tumor resection, depending upon the postoperative course, as chemotherapy can interfere with the wound-healing process and the production of cytokines at the wound site that have been shown to be critical to the healing process. This leaves the patient in a potential vulnerable setting as the tumor cells are free to divide and grow unchecked. Delays > 2 weeks postoperatively correlate with a poorer overall survival [7, 8]. Two independent retrospective studies showed that increased time from the definitive surgery to the resumption of chemotherapy was associated with an increased risk of death in patients who presented with localized osteosarcoma [7, 8]. A study from the Children's Oncology Group (COG) showed that, of 703 patients analyzed, resumption of chemotherapy ranged from 3 to 97 days following resection, with >50% of patients going >2 weeks without treatment and 22% having chemotherapy delays in excess of 21 days [8]. The times to chemotherapy resumption following surgery were dependent on several factors including the type of surgery and surgical margins. Location of the tumor (proximal versus distal extremity), tumor size (< or ≥9 cm), and % necrosis did not play a role. There was a significant decrease in overall survival in those patients that had a delay of >16 days. Patients with a delay in excess of 21 days had a 57% increased risk of death. These findings were confirmed in a more recent analysis of 77 patients with a median follow-up time of 11 years [7]. Prolonged time to the resumption of chemotherapy was once again associated with an inferior overall survival, and 75% of patients had delays beyond 21 days. Additionally concerning is that a recent study showed that the median time interval between the last neoadjuvant chemotherapy before surgery and the first dose of chemotherapy postoperatively was 50–63 days. This prolonged interval with no treatment gives the micrometastases in the lung that were not eradicated by the neoadjuvant therapy the opportunity to grow. Once a patient relapses with lung metastases, effective therapeutic options are limited as salvage chemotherapy has made no impact on long-term survival [36]. Therefore, the time a patient spends in the postoperative period without receiving chemotherapy puts the patient at significant risk. Finally, patients with a poor tumor response were found to have a significantly increased risk for relapse if there was a delay of >24 days postoperatively in the initiation of chemotherapy compared to those with a good tumor response [9]. This is the most vulnerable population as histologic response correlates with both disease-free and overall survival [9]. As chemotherapy delays increased, both populations were at higher risk for relapse and decreased survival [9]. Therefore, identifying treatments that can kill residual tumor cells in the lung which can be initiated in the immediate postoperative period because they have no adverse effect on the wound-healing process has the potential to decrease the relapse rate in both good and poor responders. Such a strategy can have a significant impact on the long-term survival of patients.

Our current investigations show that aerosol GCB results in significantly lower serum levels compared with its systemic administration. The dose used in our studies was 1/10th the systemic dose normally given to evaluate efficacy [22]. We have previously evaluated the efficacy of aerosol versus i.p. GCB against both primary OS and OS lung metastases [22]. Treatment in these studies was similar to that in the present investigations. When the primary tumor reached 130 mm^3^ and micrometastases were present in the lung, the mice were treated 3 times weekly for 3 weeks. While aerosol GCB was effective against both the primary tumor and the lung metastases, i.p. GCB at the equivalent dose was only effective against the primary tumor. GCB given i.p. was not effective against the OS lung metastases [22]. Since i.p. GCB was not effective against lung metastases, we did not evaluate the effect of systemic GCB on wound healing. Our present studies focused on evaluating the *simultaneous* efficacy of *aerosol* GCB against pulmonary metastases and its effect on wound healing. The therapeutically appropriate dose of i.p. GCB would yield even higher serum GCB levels which are more likely to interfere with the healing process. The goal of our study was to evaluate the simultaneous activity of aerosol delivery of the drug on established lung metastases in the setting of a surgical resection. Supportive of the safety and tolerability of aerosol GCB are investigations showing that there was no organ or hematologic toxicity following 5 weeks of aerosol GCB given 3 times per week [21, 22]. More importantly, our present investigations show that aerosol GCB initiated 48 hours following amputation of the limb with the primary osteosarcoma tumor inhibited the growth and eradicated established micrometastases in the lung without interfering with wound healing in the skin or bone. Aerosol GCB had no effect on the 3 phases of wound healing in the dorsal skin, tail, or bone. Cell proliferation, the number of fibroblasts and mast cells, macrophage recruitment, and the ratio of Type I to Type II macrophages in the dorsal skin and tail wounds of the mice treated with aerosol GCB were similar to those of the mice treated with aerosol PBS.

Anti-VEGF therapy has been shown to result in late wound dehiscence [37]. Aerosol GCB did not inhibit endothelial cell growth or VEGF levels in the wounds and had no effect on the production of either IL-10 or FGF-2 (bFGF). Similarly, when the wound at the amputation site was examined, treatment with aerosol GCB had no effect on CD31, VEGF, bFGF, IL-10, or the number of fibroblasts. This is the first demonstration that aerosol GCB can have a therapeutic effect on lung metastases without interfering with wound healing following surgical resection of the primary tumor, a critical process in the treatment regimen for osteosarcoma. Taken together, our data suggest that the initiation of aerosol GCB in the immediate postoperative period has the potential to eradicate lung micrometastases without fear of interfering with the postoperative healing process in the skin or the bone which can delay the resumption of the systemic chemotherapy. Eradication of lung metastases during this period when systemic chemotherapy has been suspended may prevent relapse and result in an increase in the event-free and overall survival. Patients who develop lung metastases have a 5-year survival rate of only 20% [4, 5].

## 5. Conclusion

Salvage chemotherapy has made little impact on this survival rate. Aggressive surgical removal of the metastases in combination with salvage chemotherapy only rescues about 40% of patients [6]. As the safety of aerosol GCB has already been demonstrated in adult patients [24], our data support the concept of using aerosol GCB as a bridge after tumor resection and the initiation of clinical trials using aerosol GCB in children and adolescents with osteosarcoma in the immediate postoperative period.
